# Vitamin D Supplementation and Sleep: A Systematic Review and Meta-Analysis of Intervention Studies

**DOI:** 10.3390/nu14051076

**Published:** 2022-03-03

**Authors:** Myriam Abboud

**Affiliations:** Department of Health Sciences, College of Natural and Health Sciences, Zayed University, Dubai, United Arab Emirates; myriam.abboud@zu.ac.ae

**Keywords:** vitamin D, sleep, systematic review, meta-analysis

## Abstract

Background: Vitamin D deficiency is associated with sleep disorders and poor sleep quality. Whether vitamin D supplementation (VDS) helps resolve these problems remains unclear. Objective: To systematically review the effect of VDS on sleep quantity, quality, and disorders, and perform a meta-analysis of available data. Methods: The reporting of this review followed the PRISMA statement. VDS human interventions studies that reported on sleep quality, quantity, or disorders were included. Medline, CINAHL, EMBASE, PsycInfo, the Cochrane Library, Clinicaltrials.gov, and the ICTRP were searched, in addition to the references of the included articles and previous relevant reviews, without language or time restrictions. Included studies were critically appraised, findings were narratively synthesized, and a meta-analysis was conducted. Furthermore, the overall certainty of the evidence was assessed. Results: A total of 19 studies were included (13 randomized controlled trials (RCTs), 1 opportunistic addition to an RCT, 4 pre–post studies, and 1 pre–post study analyzed as a case series); 3 RCTs were meta-analyses. The risk of bias was generally low. Pre–post studies showed a significant improvement in sleep quality with VDS. Similarly, the results of the meta-analysis revealed a statistically significant decrease in the Pittsburgh Sleep Quality Index with VDS compared with placebo (mean difference, −2.33 (95% CI, −3.09, −1.57); *p* < 0.001; I^2^ = 0%), with a moderate certainty of evidence. The results regarding the effect of VDS on sleep-related impairment, difficulty, and disorders, as well as sleepiness and restless legs syndrome, were not unanimous. Conclusions: VDS is promising in improving sleep quality; however, its effect on sleep quantity and disorders needs to be further investigated.

## 1. Introduction

Inadequate sleep is a common public health problem of significant personal and societal burden [1]. Sleep disorders such as insomnia, obstructive sleep apnea (OSA), excessive daytime sleepiness (EDS) and fatigue, sleep deprivation, and restless legs syndrome (RLS) are increasingly being diagnosed in clinical practice [2]. It is estimated that 59% of young adults suffer from a sleep disorder and do not get enough sleep [3] and only 36% of this population reports being free of sleep disturbances [4]. Inadequate sleep is an underappreciated determinant of health [5,6] and can lead to short-term and long-term consequences. In the short run, inadequate sleep may result in cognitive and motor performance impairments, which can lead to decreased quality of life and reduced productivity [7,8]. In the longer term, cumulative sleep deprivation can serve as a factor in the development and exacerbation of cardiovascular and metabolic diseases, cancer, diabetes mellitus, gastrointestinal disorders, and mental illnesses [6,9]. Thus, the economic burden of inadequate sleep is substantial, warranting urgent investment in health measures to address this issue [1].

Low vitamin D status is a prevalent condition that has been linked to a wide range of adverse health outcomes [10,11]. Growing evidence has demonstrated that vitamin D has a role in sleep regulation [12]. Specifically, vitamin D deficiency (VDD) can increase risk of sleep disorders and is associated with sleep difficulties, shorter sleep duration, and nocturnal awakenings in children and adults [13,14,15]. The exact mechanisms by which vitamin D regulates sleep are still far from being elucidated. Plausible theories include the presence of vitamin D receptors on areas of the brainstem that are known to be pacemaker cells playing an important role in sleep regulation [16,17], in addition to the potential role of vitamin D in regulating melatonin, the “sleep hormone” [18].

Hence, it is plausible that vitamin D supplementation (VDS) might have a positive effect on sleep disorders, including decreased sleep latency, improved sleep efficiency, and longer sleep duration. Preventing and managing sleep disorders or correcting them by VDS is of public health relevance, given the low cost of this intervention and its effectiveness in other therapeutic areas. Given the lack of conclusive evidence in this regard, we aim to systematically review the available literature on the effect of VDS on sleep quantity, quality, and sleep disorders, and perform a meta-analysis of the available data. 

## 2. Materials and Methods

### 2.1. Review Design

A predefined protocol registered at the OSF registries (DOI: 10.17605/OSF.IO/27BD3) was followed to conduct this review. Ethical approval was not mandatory. 

### 2.2. Criteria for Study Inclusion

Interventional studies, whether controlled or not, conducted on individuals not having diseases or receiving medication known to influence vitamin D metabolism, such as chronic kidney disease or liver disease, with or without sleep disturbances, including vitamin D supplementation in any form, dose, or frequency as intervention, and reporting on the prevalence or severity of sleep disorders [19], such as insomnia disorders, sleep-related breathing disorders, central disorders of hypersomnolence, circadian rhythm sleep–wake disorders, parasomnias, and sleep-related movement disorders, or sleep quantity or quality, were included. 

All studies extending supplementation for a minimum of 4 weeks were included in this review to ensure adequate time for the intervention to produce an effect. In addition, controlled studies involving a placebo, or a lower dose, or different form of vitamin D were included. Finally, controlled studies including a co-intervention were included if both arms of the study received the same co-intervention.

Eligible studies were those written in any language, irrespective of publication date (i.e., no language or time limit).

Exclusion criteria included non-original studies (e.g., case reports, case series, editorials, and reviews) and studies conducted on participants with conditions (e.g., chronic kidney disease) or on medications that might have an effect on vitamin D metabolism (e.g., phenytoin, phenobarbital, carbamazepine, and rifampin). Finally, studies evaluating the association between vitamin D status (hypo or hypervitaminosis D) and sleep were not included.

### 2.3. Search Strategy

The author searched Medline via Ovid, the Cumulative Index to Nursing and Allied Health Literature (CINAHL) via EBSCO, EMBASE via Ovid, APA PsycInfo via Ovid, the Cochrane Library, Clinicaltrials.gov, and the International Clinical Trials Registry Platform (ICTRP). Vitamin D supplementation and sleep were the key concepts followed in the search strategy: for each concept, Medical Subject Headings (MeSH) and keywords were recorded, whereby search terms included vitamin D, cholecalciferol, ergocalciferol or calcidol, combined with sleep or insomnia. The author did not apply any language or publication date restrictions to the search. The author also hand searched the reference lists of included articles and previous relevant reviews for eligible studies. The electronic search strategy was validated by a medical information specialist and the search strategy and results for Medline, Embase, and the Chocrane Library are available in Supplementary S1.

### 2.4. Study Selection

Studies meeting the inclusion criteria previously specified were identified by screening titles and/or abstracts from electronic scientific databases via Endnote, version X6. Full texts of potentially eligible studies were retrieved. Finally, records in the grey literature search zone were further assessed for eligibility of inclusion.

### 2.5. Data Extraction

For all eligible studies, data related to the features of the study, population groups, interventions given (type, form, and the dose of vitamin D in experimental groups, comparator, and duration), outcomes, and main findings were extracted and recorded in a data extraction form. When reported as nmol/L, the author converted serum 25OHD to ng/mL by dividing by a factor of 2.496. The author contacted the authors of some included studies to obtain additional data, when they were not reported in the published studies. 

### 2.6. Quality Assessment

The Cochrane criteria (sequence generation, allocation concealment, blinding of participants and outcome assessors, incomplete outcome data, and selective outcome reporting) were used as the tool to assess the risk of bias of RCTs included in this review [20]. Furthermore, a modified version of the Cochrane Risk of Bias tool [21] (eligibility criteria, measurement of exposure and outcome, control confounding, and follow-up) was used to assess the risk of bias of non-randomized studies. Potential sources of bias for both RCTs and non-randomized studies were graded as low, high, or unclear risk. 

The Grading of Recommendations Assessment, Development, and Evaluation (GRADE) criteria (risk of bias, inconsistency, indirectness, imprecision, and publication bias) were used to assess the overall certainty of the evidence presented using GRADE Evidence Profiles developed in the GRADEpro GDT software (www.gradepro.org; accessed on 8 November 2021).

### 2.7. Data Synthesis

A narrative composite of the study findings was provided when a meta-analysis was not feasible. Furthermore, author-recorded features of the study, population group characteristics, intervention provided, comparator, and the outcome were included in this composite.

A meta-analysis was conducted when participants, treatments, and outcomes shared similar characteristics to allow pooling. Standard meta-analyses comparing VDS with placebo were performed using RevMan version 5.4 (The Cochrane Collaboration, The Nordic Cochrane Centre). A random-effects model was used for the analysis of more than two studies. The results were reported as the mean difference with 95% confidence intervals. The I^2^ statistic was used to assess heterogeneity among different studies.

### 2.8. Quality of Reporting 

The Preferred Reporting Items for Systematic reviews and Meta-Analyses literature search extension (PRISMA-S) checklist and the PRISMA statement were followed for the literature search component [22], and the reporting of this systematic review [23].

## 3. Results

### 3.1. Search Results

**Figure 1:**
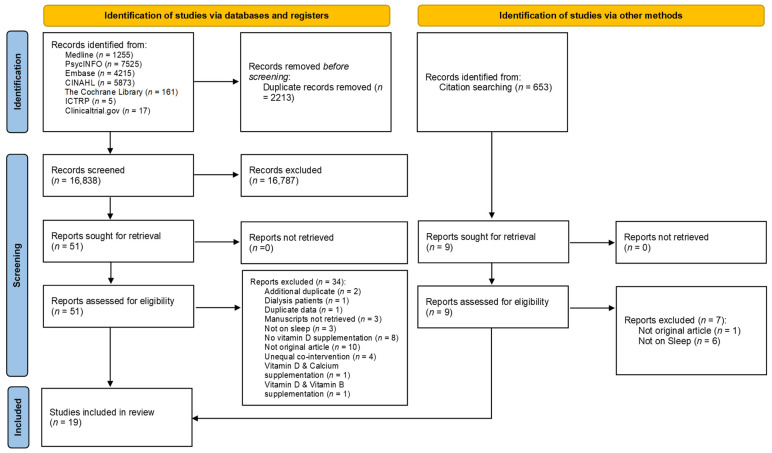
Preferred Reporting Items for Systematic Reviews and Meta-Analyses (PRISMA) diagram of study selection. ICTRP: International Clinical Trials Registry Platform.

Details of the search process are presented in Figure 1. Out of the 19,051 screened records, 19 studies were included in this systematic review. Out of these studies, thirteen were RCTs [24,25,26,27,28,29,30,31,32,33,34,35,36], one was an opportunistic addition to an established randomized, double-blind, placebo-controlled trial [37], four were pre–post studies [38,39,40,41], and one was a self-controlled before–after trial, analyzed retrospectively as a case series [42]. 

Three out of thirteen RCTs generated data that could be combined in the meta-analysis.

### 3.2. Characteristics of Included Studies

Table 1 demonstrates the characteristics of the included studies. Six of the studies were conducted in Iran [25,27,30,31,34,39], five in the USA [28,29,32,33,42], one in Ireland [26], one in New Zealand [37], one in KSA [35], one in China [36], one in Turkey [40], and one in the Netherlands [24]. The number of participants varied from 5 [31] to 18,353 [32]. Two studies were conducted in the pediatric population: children with Autism Spectrum Disorder [40] and ADHD [31], one study was conducted on overweight postmenopausal women [28], another in elderly women [39], and one on community-dwelling older people [24]. One study was conducted in patients receiving maintenance methadone treatment [25], one in active-duty warfighters [29], one on veterans with multiple areas of chronic pain and low serum 25(OH)D [42], one on adult patients with urticarial [33], one on fibromyalgia syndrome patients [30], one in patients with chronic low back pain [41], and one on patients with depression with tied anxiety symptoms [36]. Finally, one study was conducted on adults with OSA [26], one on adults with sleep disorders [27], two in patients with RLS [35,38], and one on adults with vitamin D deficiency, abdominal obesity, and symptoms of insomnia [34].

In the majority of the studies, the intervention consisted of vitamin D3 supplementation [24,26,27,28,29,32,33,34,35,37,38,42], two used vitamin D2 supplementation [40,42], and the form of vitamin D was unclear in six trials [25,30,31,36,39,41]. Only Sharifan et al. [34] assessed VDS in the form of fortified low-fat milk or low-fat yogurt. The duration of supplementation ranged from 8 weeks [27,30,31,39,41] to a median of 5.3 years [32]. The average daily dose of VDS ranged from 1000 IU [29,39] to 7142.85 IU [30,35,42]. When reported, compliance with VDS was high in all studies. The majority of the studies were placebo controlled [24,25,26,27,28,30,31,32,35,36,37]. In two studies, the comparator was no supplementation [29,39]; in one study, it was a lower dose of vitamin D [33]; in one study, it was either low-fat milk or yogurt [34]. The most assessed outcome was sleep quality, mainly using the Pittsburgh Sleep Quality Index (PSQI) [25,27,28,30,39,41,42].

### 3.3. Assessment of Risk of Bias

**Figure 2:**
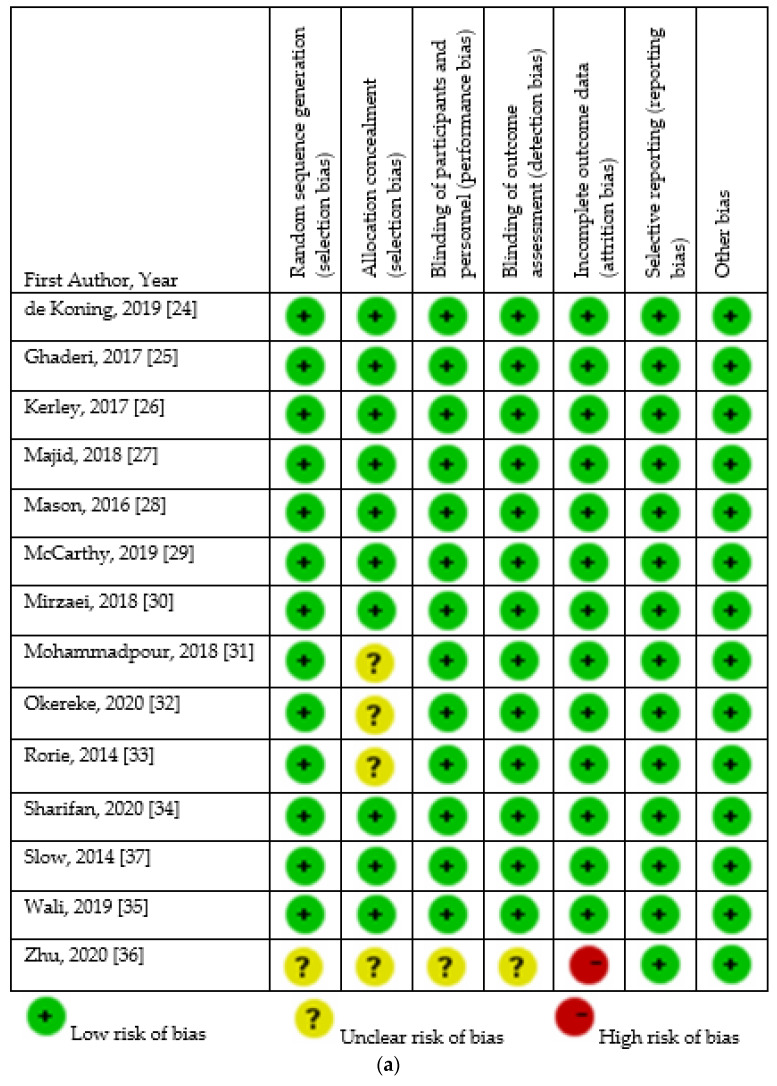

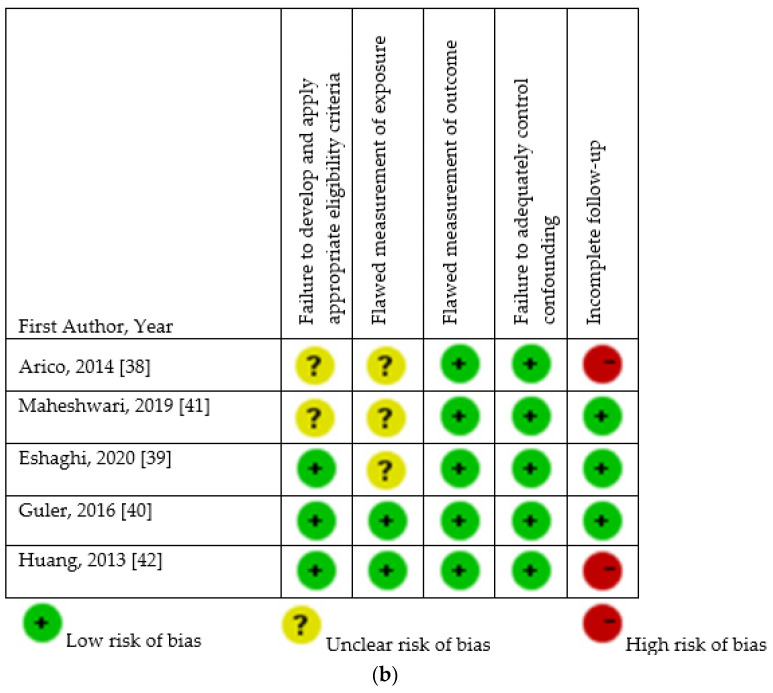
Risk of bias of included studies. (**a**). Risk of bias of included randomized controlled trials. (**b**). Risk of bias of included non-randomized studies [24,25,26,27,28,29,30,31,32,33,34,35,36,37,38,39,40,41,42].

The assessment of the risk of bias of included studies is presented in Figure 2. Regarding RCTs, the risk of bias was low, except for the study by Zhu et al. [36]. As for non-randomized trials, measurement of exposure was unclear in the studies conducted by Arico et al. [38], Eshaghi et al. [39], and Maheshwari et al. [41]. Finally, risk of bias regarding incomplete follow-up was high in the studies carried out by Arico et al. [38] and Huang et al. [42]. 

Findings from the included studies are presented in Table 2.

### 3.4. Sleep Quality

The pre–post studies by Huang et al. [42], Eshaghi et al. [39], and Maheshwari et al. [41] investigated the effect of VDS on sleep quality assessed by the PSQI. All three trials showed a significant improvement in overall sleep quality with VDS. However, the three trials were heterogeneous and did not contain ample information allowing pooling; hence, performing a meta-analysis of their results was impossible. 

The four RCTs conducted by Ghaderi et al. [25], Majid et al. [27], Mason et al. [28], and Mirzaei et al. [30] explored the effect of VDS on sleep quality assessed by the PSQI. Only three [25,27,30] provided information and had similar characteristics to allow pooling, whereas the study by Mason et al. [28] did not report on numerical outcomes, and therefore was not included in the meta-analysis. This study showed no significant change in overall sleep quality with VDS and a deterioration in total PSQI among women who repleted their vitamin D levels, concluding that VDS of 2000 IU/d may result in overall worse sleep quality for postmenopausal women with low circulating vitamin D undergoing weight loss.

**Figure 3:**
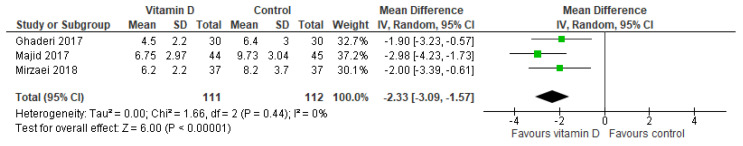
Meta-analysis of the effect of vitamin D supplementation on sleep quality assessed by the Pittsburg Sleep Quality Index. Mean differences for each study are represented by squares, and 95% Confidence Intervals are represented by the lines through the squares. The pooled mean differences are represented by diamonds. Between-study heterogeneity was assessed with the use of the I^2^ statistic [25,27,30].

As for the results of the meta-analysis of the eligible RCTs [25,27,30], the forest plot for the mean difference in the PSQI based on VDS is presented in Figure 3. The three studies included patients undergoing maintenance methadone treatment [25], people with a PSQI ≥ 5 [27], and fibromyalgia syndrome patients [30]. The duration of VDS was short (8 [27,30] to 12 weeks [25]), and the dose was either 3571.42 [25,27] or 7142.85 IU [30]. A statistically significant decrease in the PSQI in the group receiving VDS as compared with placebo was shown by the meta-analysis (mean difference, −2.33 (95% CI, −3.09, −1.57); *p* <0.001). The heterogeneity of the analysis was null (I^2^ = 0%). The overall certainty of the evidence of the meta-analysis was moderate (Supplementary S2).

### 3.5. Other Outcomes

**Disturbed sleeping**

The only pre–post study [40] investigating sleep habits and disorders in children with ASD showed that VDS may be beneficial in these patients, as well as healthy individuals with sleep disturbances.

As for RCTs, the effects of VDS on sleep-related impairment, sleep difficulty, and sleep disorders were assessed by McCarthy et al. [29], Okereke et al. [32], and Zhu et al. [36], respectively; the results were not unanimous. While McCarthy et al. [29] showed a statistically significant improvement in sleep-related impairment with VDS, Okereke et al. [32] and Zhu et al. [36] did not report on such findings. In both RCTs, there were non-significant differences in likelihood of sleep problems with VDS compared with placebo after controlling for confounding variables).

**Sleepiness**

Two RCTs assessed the effect of VDS on sleepiness using different tools [26,34]. The study of Kerley et al. [26], which was conducted on patients with OSA, did not report any difference in sleepiness between the group receiving VDS and those receiving placebo. In contrast, the study by Sharifan et al. [34], which was conducted on patients with insomnia, showed a beneficial effect of vitamin D3-fortified low-fat milk on insomnia symptoms compared with unfortified milk. No effect was detected with vitamin D3-fortified low-fat yogurt compared with unfortified one.

**RLS**

The pre–post study conducted by Arico et al. [38] found that long-term VDS (6 months) decreased RLS severity in a small population (5 patients). In contrast, the RCT by Wali et al. [35] found no effect of VDS on severity of RLS compared with placebo.

### 3.6. Sleep Problems as Adverse Events of VDS

Sleep problems as an adverse event of VDS were assessed in the two RCTs conducted by Mohammadpour et al. [31], and de Koning et al. [24]. Both studies showed no significant difference in sleep problems with VDS versus placebo in children with ADHD or community-dwelling people with depressive symptoms, respectively. 

## 4. Discussion

This systematic review and meta-analysis investigated the effect of VDS on sleep quantity and quality, and sleep disorders. VDD is an emerging risk factor for suboptimal sleep and sleep disorders [12,15,43]. Such an association has been observed in several healthy and ill populations including factory workers, older community-dwelling men, hemodialysis patients, and pregnant women [12]. Specifically, through a meta-analysis of observational studies involving 9397 participants, Gao et al. found that participants with VDD had increased odds of sleep disorders and poor sleep quality by 1.5 fold, short sleep duration by 1.75 fold, and sleepiness by 1.36 fold. They also provided evidence that serum 25(OH)D below 20 ng/mL could significantly heighten the odds of unhealthy sleep [15]. Similarly, through a meta-analysis of observational studies conducted on 1864 subjects with sleep disorders and 1340 control participants, Yan et al. [43] found that the average serum vitamin D concentration in the group with sleep disorders was 0.75 ng/mL lower than that in the control group [43].

The association between VDS and sleep regulation is biologically plausible and worth investigation given its clinical and public health relevance. Nevertheless, we found a limited number of human interventional studies—especially RCTs—and most of the included studies focused on sleep quality compared with other sleep-related outcomes. The evidence from included studies was promising regarding the effectiveness of VDS on enhancing sleep quality; nevertheless, studies investigating sleep quantity and sleep disorders were scarce, heterogeneous in terms of included populations and methodologies, and their findings were not unanimous, preventing generating a solid conclusion. Hence, our results suggest that VDS is promising in improving sleep quality; however, its effect on sleep quantity and disorders needs to be further investigated. 

Although the exact physiological mechanisms between vitamin D and sleep regulation have not yet been fully uncovered, several direct and indirect mechanisms have been suggested [12]. One potential mechanism is the extensive presence of vitamin D receptors in many parts of the brain, specifically areas that affect sleep [44]. Another theory involves the expression enzymes involved in vitamin D activation and degradation (25-hydroxylase and 1- hydroxylase and 24-CYP24A1) in areas in the brain known to be involved in sleep regulation including the supraoptic and paraventricular nuclei within the hypothalamus and the substantia nigra [44,45]. Another plausible theory considers the effect of sunlight. It is well known that vitamin D levels are regulated by exposure to sunlight and since sunlight also affects the circadian rhythm, it is highly plausible to assume that there is a link between those factors [46,47,48,49]. Furthermore, the production of melatonin—a hormone involved in the regulation of circadian rhythms and sleep—is regulated by vitamin D; thus, impaired vitamin D levels could decrease melatonin levels, potentially leading to sleep disorders [50,51]. One final plausible mechanism is that vitamin D, as an immunomodulatory molecule, plays a role in downregulating inflammatory markers that are involved in sleep regulation such as tumor necrosis factor α (TNF-α), cytokines and prostaglandin D2. In the case of VDD, such inflammatory markers would be raised, negatively affecting sleep [52,53]. All of these factors may explain our findings regarding the beneficial effect of VDS on sleep quality. 

The only study involving patients with OSA was conducted by Kerley et al. [26]. VDD is a common finding in OSA patients compared with non-apneic subjects, and vitamin D levels were shown to be inversely correlated with the severity of OSA [54]. This association is likely to be mediated by complex pathogenetic mechanisms, such as immune system modulation, myopathy, and inflammation; nevertheless, these mechanisms are not fully understood yet. Additionally, this relationship seems to be confounded by numerous factors, such as obesity [54]. Kerley et al. [26] did not find a beneficial effect of VDS on improving sleepiness in this patient population. This might be due to the small sample of the trial and to the fact that 90% of the VDS group were stable on continuous positive airways pressure, which may have diluted any potential benefit of VDS [26].

Our findings concerning the effectiveness of VDS on RLS were contradictory [35,38]. VDD may play a role in this movement disorder through its link with dopaminergic dysfunction. Nevertheless, to date, the causality between VDD and RLS is only hypothesized. This null effect, despite the significant improvement in vitamin D levels in the intervention group, suggests that VDS may not have a therapeutic effect in RLS, although it may contribute to the pathophysiology of the syndrome [35]. The authors of this study argue that in RLS, vitamin D levels in the brain are more important than in the blood. Accordingly, the improvement in serum levels with VDS may not have sufficiently affected vitamin D levels in the brain [35]. 

It should be noted that vitamin D levels in response to supplementation depend on three factors, dose, frequency and interval [55]; accordingly, our findings could be interpreted in light of this fact. For example, the RCTs that found a beneficial effect of VDS on sleep disorders were conducted over a short period of time, i.e., 10 weeks in the study by McCarthy et al. [29], and 12 weeks in the studies by Rorie et al. [33] and Sharifan et al. [34]. In contrast, RCTs reporting no significant improvement in sleep disorders with VDS were those conducted over a long duration, i.e., 6 months in the study by Zhu et al. [36], 18 months in the study by Slow et al. [37], and a median follow-up duration of 5.3 years in the study by Okereke et al. [32]. This observation, although it could be attributed to seasonality, raises queries on whether extended supplementation with vitamin D may not always result in better outcomes. 

Finally, human circadian rhythms—sleep and wakefulness cycles—are synchronized by environmental signals, especially light and dark intervals through sunlight [48]. Although intentional sun exposure could hence be recommended to enhance sleep and vitamin D levels, it remains highly challenging to titrate one’s exposure, besides the documented negative side effects of ultraviolet irradiation. 

### Strengths and Limitations

Our study has numerous strengths. First, we followed a systematic approach in our search and analysis, using a highly sensitive search strategy, and followed recommended reporting approaches for the review [23] as well as the search strategy [22]. Second, we contacted the authors of some included studies to obtain additional data when they were not reported in the published studies. Unfortunately, we did not receive feedback from all authors. Finally, the risk of bias of the majority of included studies was low. 

Nevertheless, the current analysis had some limitations. First, we were limited to study level rather than individual-level data, which would have been more accurate than the overall mean change in sleep. Second, there were variabilities between included studies, which complicates the comparisons as well as the interpretation of our results, especially in the study populations, the outcomes assessed, and the assessment methods. Third, some papers did not provide crucial information such as the form of vitamin D, the levels of vitamin D at the end of the study, the daily dose equivalent, the assessment method of vitamin D levels, nor compliance with VDS. Although we contacted respective authors, we could not obtain the needed information in some instances. This would have enabled us to better interpret our findings. Fourth, while we tried to make the literature search as exhaustive as possible, pertinent studies might have been missed; a common limitation to systematic reviews. Furthermore, we did not have access to some potentially eligible studies for full-text screening. Fifth, given the small number of studies investigating the effect of VDS on sleep disorders and their heterogeneity, we could not perform a meta-analysis of their findings and our conclusion remains limited to a qualitative synthesis. Finally, repeat screening, selection of studies, data extraction, and quality assessment was not possible.

## 5. Conclusions

In conclusion, the evidence presented in this review suggests a beneficial role of VDS in enhancing sleep quality. These results remain to be interpreted with caution given the limited number of included RTCs and their relatively small sample size. Nevertheless, the positive effects of such supplementation could be considered in clinical practice, especially in the context of beneficial skeletal [56] and pleiotropic extraskeletal effects [57,58] of vitamin D, as well as the relatively limited cost of VDS. As we could not find enough studies assessing the effect of VDS on sleep disorders, OSA, sleepiness, and RLS, this remains to be explored in future adequately powered, high-quality RCTs.

## Figures and Tables

**Table 1:** Characteristics of included studies.

First Author, Country	Study Design	Study Population	Age %Male	Control	Co-Intervention	Intervention Duration Daily Dose Equivalent	Compliance	Vitamin D Assessment
*Randomized, controlled trial*								
Sleep quality								
Ghaderi [25], Iran	Randomized, double-blind, placebo-controlled trial	68 patients with maintenance methadone treatment referred to a clinic (30 completed the trial: I: n = 30; C: n = 30)	Age: Range: 25–70 Mean ± SD: I: 40.1 ± 9.2 C: 42.5 ± 8.9 %Male: NR	Placebo, capsule	None	Vitamin D (unclear): capsules 12 weeks 3571.42 IU	>90% in both groups	25(OH)D: commercial ELISA kit
Maheshwari [41], NR	Pre–post study	40 patients diagnosed with chronic low back pain	Age: Range: 25–65 %Male: 60%	Self	None	Vitamin D (unclear) 8 weeks NR	NR	NR
Majid [27], Iran	Randomized, double-blind, placebo-controlled trial	93 people with sleep disorders (PSQI ≥ 5), but without sleep disorders, not using sleep medications, recruited from a hospital (89 completed the trial: I: n = 44; C: n = 45)	Age: Range: 20–50 Mean ± SD: I: 37.9 ± 9.50 C: 35.5± 10.00 %Male: I: 25.00; C: 22.22	Placebo, capsule	None	Vitamin D3: edible pearls 8 weeks 3571.42 IU	I: 97.82% C: 97.87%	25(OH) D: Immunodiagnostic Systems kit
Mason [28], USA	Randomized, double-blind, placebo-controlled trial	218 overweight (BMI ≥25 kg/m2) postmenopausal women, with serum 25(OH)D ≥10 to <32 ng/mL undergoing weight loss I: n = 109; C: n = 109	Age: Range: 50–75 Mean ± SD: 59.6 ± 5.1 %Male: 0%	Placebo, gel capsules	Lifestyle-based weight-loss program including a reduced-calorie diet (1200–2000 kcal/day, <30% daily energy intake from fat) and 225 min/week of moderate-to-vigorous aerobic activity	Vitamin D3, gel capsules 12 months 2000 IU	I: 98% C: 96%	25(OH)D: DiaSorin LIAISON 25-OH Vitamin D total assay
Mirzaei [30], Iran	Randomized, double-blind, placebo-controlled trial	74 fibromyalgia syndrome patients (according to the American College of Rheumatology criteria) with 25(OH)D < 30 ng/mL, recruited from a rheumatology center	Age: Range: 20–70 Mean ± SD: Total: 41.6 ± 10.5 I: 42.1 ± 10.8 C: 41 ± 10.3 %Male: 0%	Placebo	Trazodone 25 mg at bedtime	Vitamin D (unclear) 8 weeks 7142.85 IU	NR	25(OH)D: commercial radioimmunoassay kit
Other outcomes								
McCarthy [29], Pacific Northwest	Randomized, double-blind, controlled trial	152 active-duty warfighters, divided into no-treatment group (C) (≥ 30 ng/mL) or treatment group (I) (<30 ng/mL) (131 completed the trial)	Age: Mean ± SD: I: 31.6 ± 8.2 C: 32.8 ± 10.5 %Male: 61.8%	No supplementation	None	Vitamin D3, gel capsules 12 weeks I1: 1000 IU I2: 5000 IU	I1: 84.8% I2: 87.9% C: not required to take pills	25(OH)D: electrochemiluminescence
Okereke [32], USA	Randomized, double-blind, placebo-controlled trial	Participants aged ≥50 years in the VITAL-DEP (Vitamin D and Omega-3 Trial-Depression Endpoint Prevention) ancillary study to VITAL, a randomized clinical trial of cardiovascular disease and cancer prevention, without clinically relevant depressive symptoms at baseline I: n = 9181; C: n = 9172	Age: Mean ± SD: Total: 67.5 ± 7.1 I: 67.5 ± 7.0 C: 67.4 ± 7.1 %Male: Total: 51% I: 50.6%; C: 51.1%	Placebo	None	Vitamin D3, pills Median follow-up: 5.3 years 2000 IU	Adherence rates (taking at least two-thirds of pills as assigned) I: Year 1: 94.8% Year 2: 92.2% Year 3: 91.5% Year 4: 91.4% Year 5: 90.6% C: Year 1: 94.6% Year 2: 92.1% Year 3: 91.0% Year 4: 90.5% Year 5: 89.8%	25(OH)D: radioimmunoassay
Rorie [33], USA	Randomized, double-blind, controlled trial	42 adult patients with physician-diagnosed chronic urticaria, recruited from tertiary care clinics I: *n* = 21; C: *n* = 21	Age: I: Mean: 43.9; Range: 20–72 C: Mean: 43.1; Range: 19–79 %Male: I: 14.2%; C: 28.5%	Low-dose vitamin D3 (600 IU/d)	10 mg of cetirizine twice daily and increase to 4 times daily as needed, 150 mg of ranitidine twice daily, and 10 mg of montelukast daily. In addition to rescue prednisone use for intolerable or uncontrolled symptoms	Vitamin D3, pills (high dose) 12 weeks 4000 IU	Excellent compliance (1 subject in the low vitamin D3 group showed <80% compliance)	25(OH)D: Tandem mass spectroscopy
Zhu [36], China	Randomized, placebo-controlled trial	158 patients with with 25(OH)D ≤ 75 nmol/L and depression with tied anxiety symptoms recruited through advertisements, assessed by the Mini-International Neuropsychiatric Interview to confirm the diagnosis of major depressive disorders (106 completed the trial: I: *n* = 62; C: *n* = 44)	Age: Mean ± SD I: 46.3 ± 9.7 C: 43.3 ± 13.7 %Male: I: 29%; C: 22.7%	Placebo	Fish oil (current intake)	Vitamin D (unclear), per os 6 months 1600 IU	NR	25(OH)D: commercial radioimmunoassay kit
Kerley [26], Ireland	Randomized, double-blind, placebo-controlled trial	26 Caucasian adults with OSA recruited from a sleep clinic (19 completed the trial: 15 on CPAP therapy and 4 CPAP naïve: I: *n* = 10; C: n = 9)	Age: I: Range: 42–71 Mean ± SD: 56 ± 10 C: Range: 32–68 Mean ± SD: 52 ± 13 %Male: I: 89%; C: 60%	Placebo, capsule	None	Vitamin D3: Capsule 15 weeks 4000 IU	93% in both groups	Total 25(OH)D: Architect 25(OH)D CMIA
Sharifan [34], Iran	Randomized, triple-blind, placebo-controlled trial	29 staff and students of a university, aged 30–50 years, with vitamin D deficiency (<30 ng/mL), abdominal obesity (waist circumference (≥94 cm for men and ≥80 cm for women), and symptoms of insomnia (according to validated Insomnia Severity Index) Milk: I: *n* = 8; C: *n* = 8 Yogurt: I: *n* = 7; C: *n* = 6	Age: Mean ± SD: 43.2 ± 6.59 Milk group: I: 39.5 ± 6.23; C: 44.5 ± 5.63 Yogurt group: I: 47.42 ± 6.8; C: 41.5 ± 5.99 %Male: Milk group: I: 20%; C: 80% Yogurt group: I: 57.1%; C: 42.8%	Simple low-fat milk (200 mL/day) Simple low-fat yogurt (150 g/day)	None	I1: Vitamin D3-fortified low-fat milk containing 1500 IU Nan I2: Vitamin D3-fortified low-fat yogurt 10 weeks 1500 IU	NR	25(OH)D: commercial ELISA kits
Wali [35], KSA	Randomized, double-blind, placebo-controlled trial	35 patients with primary RLS identified based on the RLS diagnostic criteria of the IRLSSG and recruited from the Sleep Medicine and Research Center I: *n* = 17; C: *n* = 18	Age: Mean ± SD I: 42.7 ± 4.7 C: 42.4 ± 5.5 %Male: I: 64.7%; C: 72.2%	Placebo	None	Vitamin D3, per os 12 weeks 7142.85 IU	100%	NR
Sleep problems as adverse events of VDS								
de Koning [24], the Netherlands	Randomized, double-blind, placebo-controlled trial	155 community-dwelling older people, aged 60–80 years, recruited from the general population or through general practitioners, with depressive symptoms, and serum 25(OH)D between 15 and 50 nmol/L during October–March or between 15 and 70 nmol/L during April–September I: *n* = 77; C: *n* = 78	Age: Median [IQR] I: 67.8 [65.4–71.7] C: 67.3 [63.4–72.0] %Male: I: 41.6%; C: 43.6%	Placebo	Calcium tablet of 500 mg/day in case of <2 dairy consumptions/day Participants were allowed to take a (multi)VDS with a maximum of 400 IU/day in addition to the study tablets	Vitamin D3, tablet 12 months 1200 IU	87.10%	25(OH)D: liquid chromatography followed by tandem mass spectrometry
Mohammadpour [31], Iran	Randomized, double-blind, placebo-controlled trial	62 children with ADHD (based on DSM-IV criteria), aged 5–12 years, referred from psychiatric centers, without psychiatric nor neurologic comorbidities (54 completed the trial: I: *n* = 25; C: *n* = 29)	Age: Mean ± SD: Total: 7.87 ± 1.61 I: 7.70 ± 1.77 C: 8.03 ± 1.44 %Male: I: 71%; C: 77.4%	Placebo	Methylphenidate	Vitamin D (unclear), tablet 8 weeks 2000 IU	100%	25(OH)D3: chemiluminescence
*Opportunistic addition to a randomized, controlled trial*								
Other outcomes								
Slow [37], New Zealand	Opportunistic addition to an established randomized, double-blind, placebo-controlled trial	322 healthy adults already participating in the vitamin D and acute respiratory infections study (VIDARIS) staff or students recruited from a University. (308 completed the trial: I: *n* = 147; C: *n* = 146)	Age: Range: 18–67 Mean ± SD: I: 47 ± 10 C: 48 ± 10 %Male: I: 25%; C: 25%	Placebo	None	Vitamin D3, per os 18 months 6557.37 for 2 months, then 3278.68 IU	NR	NR
*Pre–post study*								
Sleep quality								
Eshaghi [39], Iran	Pre–post study	42 elderly women referred to a sports counseling center, with a PSQI > 11, without sleep apnea, not smoking, and not taking hypnotic drugs (36 completed the trial)	Age: Range: 60–70 %Male: 0%	No supplementation (habitual daily activities)		Vitamin D (unclear) 8 weeks 1000 IU	NR	NR
Other outcomes								
Guler [40], Turkey	Pre–post study	Cases: 60 patients with ASD according to DSM V criteria, aged between 4 and 10 years Controls: 60 age- and sex-matched apparently healthy children	Age: Mean ± SD: Cases: 7.10 ± 1.50 Controls: 6.93 ± 1.59 %Male: Cases: 73.3%; Controls: 65%			Vitamin D2 3 months Vitamin D according to deficiency level: I1: Participants with 25(OH)D: 20–29 ng/mL: 5000 IU I2: Participants with 25(OH)D < 20 ng/mL: 7142.86 IU	NR	25(OH)D: radioimmunoassay using commercial kits
Arico [38], Italy	Pre–post study	5 patients with RLS recruited from a sleep center	Age: NR %Male: 0%	Self	None	Vitamin D3 (unclear) 6 months NR	NR	NR
*Pre–post study, analyzed retrospectively as a case series*								
Sleep quality								
Huang [42], USA	Pre–post study, analyzed retrospectively as a case series by medical record review	46 veterans with multiple areas of chronic pain and low serum 25(OH)D (<30 ng/mL) at baseline recruited from a major Veterans Affairs Medical Center, divided into vitamin D (1) INS: 25(OH)D: 20–29 ng/mL; and (2) DEF: 25(OH)D: <20 ng/mL (28 completed the trial: INS: *n* = 15; DEF: *n* = 13)	Age: Mean ± SD: 46.2 ± 10.8 %Male: 64.3%	Self	None	INS: Vitamin D3: per os DEF: Vitamin D2: per os 12 weeks INS: 1200 IU DEF: 7142.85 IU	NR	25(OH)D: liquid chromatography–mass spectrometry assay

I: intervention; C: control; PSQI: Pittsburgh Sleep Quality Index; OSA: obstructive sleep apnea; NR: not reported; INS: insufficient; DEF: deficient; VDS: vitamin D supplementation; 25(OH)D: 25-hydroxyvitamin D; SD: Standard Deviation; IU: International Unit; CPAP: Continuous Positive Airways Pressure; CMIA: Chemiluminescent Microparticle Immunoassay; ELISA: Enzyme-Linked Immunosorbent Assay; RLS: restless legs syndrome; IRLSSG: International Restless Legs Syndrome Study Group; DSM: Diagnostic and Statistical Manual of Mental Disorders; ADHD: Attention Deficit Hyperactivity Disorder; IQR: Interquartile Range; ASD: Autism Spectrum Disorder.

**Table 2:** Results of included studies.

First Author, Country	Outcomes Evaluated and Assessment	Baseline 25OHD Level	Endline 25OHD Level	Baseline Outcomes	Endline Outcomes	Conclusion
*Randomized, controlled trial*						
Sleep quality						
Ghaderi [25], Iran	Sleep quality: PSQI	I: 13.9 ± 4.5 C: 13.5 ± 4.5 (NS difference between I and C)	I: 22.0 ± 7.5 (sig. increase) C: 13.1 ± 5.9	I: 6.0 ± 2.3 C: 6.6 ± 2.2	I: 4.5 ± 2.2 (sig. decrease) C: 6.4 ± 3.0	PSQI sig. decreased in VDS I group compared with C group (−1.5 ± 2.2 vs. −0.2 ± 2.3)
Maheshwari [41], NR	Sleep quality: PSQI	NR	NR	NR	t-test: 2.965; CI: 1.8312–6.8341; *p* = 0.004 (sig. differences before and after VDS)	VDS improves sleep in patients with chronic low back pain
Majid [27], Iran	Sleep quality: PSQI Sleep duration Sleep latency Sleep efficiency: real sleep duration from the whole time passed in bed Sleep disturbances Use of sleep medications Daytime dysfunction: experiencing problems resulted by sleeplessness Subjective sleep quality	I: 25.00 ± 8.95 C: 27.60 ± 8.30 (NS difference between I and C)	I: 37.69 ± 12.25 C: 27.97 ± 7.46 (sig. increase in I, and sig. difference between I and C)	PSQI (score) (NS difference between I and C) I: 9.45 ± 2.44 C: 10.51 ± 3.14 Sleep duration (hour) (sig. higher in I compared with C) I: 5.83 ± 1.15 C: 5.22 ± 1.54 Sleep latency (minute) (NS difference between I and C) I: 49.88 ± 38.99 C: 65.00 ± 47.54 Sleep efficiency (%) (NS difference between I and C) I: 82.58 ± 9.93 C: 78.20 ± 12.90 Sleep disturbances (score) (NS difference between I and C) I: 1.23 ± 0.47 C: 1.40 ± 0.78 Use of sleep medications (time per week) (NS difference between I and C) I: 2.07 ± 1.92 C: 0.77 ± 1.02 Day time dysfunction (score) (NS difference between I and C) I: 1.57 ± 0.99 C: 1.17 ± 0.93 Subjective sleep quality (score) (NS difference between I and C) I: 1.68 ± 0.77 C: 1.57 ± 0.62	PSQI (score) (sig. lower in I compared with C) I: 6.75 ± 2.97 (sig. decrease) C: 9.73 ± 3.04 Sleep duration (hour) (sig. higher in I compared with C) I: 6.50 ± 1.49 C: 5.21 ± 1.44 Sleep latency (minute) (sig. lower in I compared with C) I: 33.18 ± 27.91 C: 58.57 ± 36.81 Sleep efficiency (%) (NS difference between I and C) I: 86.97 ± 11.39 (sig. decrease) C: 80.89 ± 11.46 Sleep disturbances (score) (NS difference between I and C) I: 1.14 ± 0.46 (NS) C: 1.41 ± 0.65 (NS) Use of sleep medications (time per week) (NS difference between I and C) I: 1.07 ± 0.94 (sig. decrease) C: 1.20 ± 0.99 Day time dysfunction (score) (NS difference between I and C) I: 0.70 ± 0.96 (sig. decrease) C: 0.75 ± 0.98 Subjective sleep quality (score) (sig. lower in I compared with C) I: 1.18 ± 0.62 (sig. decrease) C: 1.46 ± 0.58	Reduced PSQI (improved sleep score), reduced sleep latency, increased sleep duration, and subjective sleep quality with VDS. NS difference in sleep efficiency, sleep disturbances, and use of sleep medications
Mason [28], USA	Sleep quality: PSQI	NR	NR	NR	NR	NS change in overall sleep quality between VDS I and C groups A greater magnitude of change in serum 25(OH)D was associated with an increased need to take medications to sleep and overall worse sleep quality Deterioration in total PSQI among women who became vitamin D replete (≥32 ng/mL) compared with those who remained <32 ng/mL (despite VDS) VDS of 2000 IU/d may result in overall worse sleep quality for postmenopausal women with low circulating vitamin D undergoing weight loss
Mirzaei [30], Iran	Sleep quality: PSQI	I: 11.4 ± 6.7 C: 13.4 ± 7.3	I: 33.5 ± 12.2 (sig. higher in I compared with C) C: 13.3 ± 7.2	I: 10 ± 3.3 C: 10.75 ± 4.4	I: 6.2 ± 2.2 C: 8.2 ± 3.7 (sig. lower in I compared with C)	Considerable improvements were observed in the PSQI score of the both study groups; yet there was a sig. greater decrease in mean PSQI score in the I compared with C group
Other outcomes						
McCarthy [29], Pacific Northwest	Sleep-related impairment: Questions from the National Institutes of Health Patient-Reported Outcomes Measurement Information System	I1: 22.2 ± 5.0 I2: 22.9 ± 4.7 C: 37.8 ± 5.6	I1: 30.80 ± 10.0 I2: 40.15 ± 7.5 (sig. higher in I2 compared with I1 and C) C: 34.46 ± 9.9	I1: 53.0 ± 7.0 I2: 48.3 ± 9.5 C: 51.5 ± 7.0	I1: 49.5 ± 9.5 I2: 45.2 ± 8.4 C: 49.3 ± 8.2	Statistically significant improvements seen across groups and over time
Okereke [32], USA	Sleep difficulty (sleep problems) as specific depressive feature (item-level symptom) from the 8 item Patient Health Questionnaire depression scale: Trouble falling or staying asleep, or sleeping too much	25(OH)D < 20 ng/mL I: 11.0% C: 12.3% Mean ± SD I: 31.2 ± 9.8 C: 31.1 ± 10.0	NR	NR	NR	NS differences in likelihood of sleep problems in the I compared with C group Adjusted differences in change in likelihood of PHQ-8 item-level symptoms, comparing vitamin D3 to Placebo: Sleep problems: Likelihood ratio: 95% CI: 1.00 (0.89–1.12) Analyses were from repeated measures logistic regression models, with follow-up time modeled as an indicator; models were controlled for age, sex, and n-3 fatty acid randomization group. Results show likelihood ratios and 95% confidence intervals (95% CIs), which reflect differences in the change in likelihood of burden from each PHQ-8 item-level symptom, comparing vitamin D3 to placebo treatment group. Differences reflect the average effect over all follow-up times since baseline
Rorie [33], USA	Nights of hives and sleep interference: from the Urticaria Symptom Severity scores	Mean(SE) C: 37.1(3.4) I: 28.8(2.2)	Mean(SE) C: 35.8(2.3) I: 56.0(3.9) (sig. higher in I compared with C)	NR	NR	Beneficial trends for sleep quality and towards decreased interference with sleep were observed with high vitamin D3
Zhu [36], China	Sleep disorder: NR	I: 15.66 ± 4.20 C: 16.86 ± 5.04 (NS difference between I and C group)	NR	NR	NR	Between-group linear mixed-model analysis showed sig. decrease in Sleep disorder (β: −0.588; 95% CI: −1.061,−0.115), that was rendered NS after controlling for confounding variables (β: −0.355; 95% CI: −0.963,0.227)
Kerley [26], Ireland	Sleepiness: ESS	I: 13.38 ± 4.64 C: 16.58 ± 8.81 (NS difference between I and C)	I: 40.38 ± 15.98 (sig. increase) C: 17.22 ± 8.57	I: 11.00 ± 5.00 C: 10.00 ± 6.00 (NS difference between I and C)	I: 6.00 ± 2.00 C: 7.00 ± 5.00 (NS difference between I and C)	No difference in ESS between the VDS I group and C group
Sharifan [34], Iran	Changes in sleepiness symptoms: Insomnia Severity Index	Milk I: 15.03 ± 3.91 C: 14.9 ± 7.34 (NS difference between I and C group) Yogurt I: 15.82 ± 4.09 C: 16.72 ± 2.96 (NS difference between I and C group)	Milk: I: 18.57 (sig. increase compared with baseline) C: 14.66 Yogurt: I: 19.93 (sig. increase compared with baseline) C: 16.26 (SD not reported)	Milk: I: 18.5 ± 3.33 C: 17.25 ± 2.34 Yogurt: I: 13.28 ± 5.12 C: 13 ± 3.54	Milk I: 13.62 ± 3.29 (sig. increase compared with baseline) C: 16.5 ± 4.02 (NS difference compared with baseline) Yogurt I: 17.57 ± 13.28 (NS difference compared with baseline) C: 16.66 ± 1.36 (NS difference compared with baseline)	Fortified low-fat milk containing 1500 IU vitamin D3 can improve insomnia symptoms
Wali [35], KSA	RLS severity: IRLSSG rating scale	I: 17.06 ± 12.6 C: 22.95 ± 16.98 (NS difference between I and C group)	I: 6.09 ± 15.38 (sig. higher in I compared with C group) C: 21.23 ± 13.74	Total I: 14.60 ± 4.5 C: 16.11 ± 6.2 In DEF patients I: 14.82 ± 5.2 C: 16.81 ± 6.3	Total: I: 14.5 ± 08.2 (NS difference compared with baseline) C: 10.3 ± 11.1 (sig. decrease compared with baseline) In DEF patients I: 13.8 ± 8.9 (NS difference compared with baseline) C: 7.8 ± 13.9 (sig. decrease compared with baseline)	NS difference in RLS severity score with VDS suggesting that VDS does not improve RLS symptoms
Sleep problems as adverse events of VDS						
de Koning [24], the Netherlands	Sleep problems as adverse event: registered by telephone or face-to-face contact	median [IQR] I: 18.42 [13.02–22.83] C: 17.68 [14.42–22.13] (significance NR)	mean ± SD I: 34.05 ± 6.41 C: 17.22 ± 7.21 (sig. difference between I and C)	NA (sleep problems as adverse event of VDS)	I: *n* = 1 C: *n* = 5 (NS difference between I and C)	NS difference in sleep problems as adverse effect of VDS in community-dwelling people with depressive symptoms
Mohammadpour [31], Iran	Sleep problems as adverse events: questionnaire	mean ± SD I: 15.792 ± 5.259 C: 12.979 ± 5.804 (NS difference between I and C)	mean ± SD: I: 34.63 ± 9.54 (sig. increase between baseline and endline) C: 11.22 ± 5.11 (NS difference between baseline and endline) Sig. difference between I and C	NA (sleep problems as adverse event of VDS)	Rate I: 4 C: 1 (NS difference between I and C)	NS difference in sleep problems as adverse effect of VDS as adjunctive therapy in children with ADHD
*Opportunistic addition to a randomized, controlled trial*						
Other outcomes						
Slow [37], New Zealand	Specific questionnaire about disruptions in sleeping patterns as a consequence of the earthquake on the 22nd February 2011	I: 29.24 ± 8.81 C: 28.44 ± 8.81	NR	NR	I: 79% C: 70% (NS difference between I and C)	VDS did not reduce the adverse impact of earthquakes in healthy adults
*Pre–post study*						
Sleep quality						
Eshaghi [39], Iran	Sleep quality: PSQI	NR	NR	Mean ± SD I: 12.55 ± 1.01 C: 12.55 ± 1.01	Mean ± SD I: 10.11 ± 1.26 (significant difference compared with baseline) C: 12.44 ± 0.88 (contradictory evidence regarding C group between written (NS difference) and tabulated (sig. difference) results)	Sleep quality sig. improved by 19.1% with VDS
Other outcomes						
Guler [40], Turkey	Sleep habits and disorders: short version of the CSHQ with a higher score reflecting more disturbed sleep behavior	Cases: 25(OH)D < 10 ng/mL: 23.3% 25(OH)D: 10–30 ng/mL: 45% 25(OH)D > 30 ng/mL: 31.7% mean ± SD: 25.58 ± 10.31 Controls: 25(OH)D < 10 ng/mL: 23.3% 25(OH)D: 10–30 ng/mL: 38.3% 25(OH)D > 30 ng/mL: 38.3% mean ± SD: 25.35 ± 9.92 (NS difference between Cases and Controls) In DEF participants: 25(OH)D: Cases: 19.68 ± 6.22 Controls: 19.21 ± 7.35	Cases: 25(OH)D < 10 ng/mL: 0% 25(OH)D: 10–30 ng/mL: 11.7% 25(OH)D > 30 ng/mL): 88.3% mean ± SD: 37.27 ± 6.51 Controls: 25(OH)D < 10 ng/mL: 0% 25(OH)D: 10–30 ng/mL: 6.7% 25(OH)D > 30 ng/mL: 93.3% mean ± SD: 37.15 ± 6.78 (NS difference between Cases and Controls) In DEF participants: 25(OH)D: Cases: 37.26 ± 7.34; sig Controls: 39.13 ± 7.74; sig	CSHQ total score Cases: <41: 21.7%; ≥41: 78.3% Controls: <41: 66.7%; ≥41: 33.3% mean ± SD total sleep time (hours) Cases: 8.10 ± 0.97 Controls: 9.24 ± 0.89 In DEF participants Total score Cases: 52.05 ± 8.24 Controls: 42.00 ± 4.78 Bedtime resistance Cases: 11.24 ± 2.49 Controls: 7.38 ± 1.01 Sleep-onset delay Cases: 2.32 ± 0.79 Controls: 1.86 ± 0.89 Sleep duration Cases: 5.41 ± 0.92 Controls: 4.41 ± 0.98 Sleep anxiety Cases: 7.73 ± 2.59 Controls: 4.41 ± 0.80 Night wakings Cases: 5.59 ± 1.61 Controls: 4.11 ± 1.39 Parasomnias Cases: 9.80 ± 2.52 Controls: 8.51 ± 1.76 Sleep-disordered breathing Cases: 3.63 ± 0.73 Controls: 3.59 ± 0.90 Daytime sleepiness Cases: 10.44 ± 1.84 Controls: 9.89 ± 1.85 Total sleep time Cases: 8.16 ± 0.89 Controls: 9.11 ± 0.89	CSHQ total score Cases: <41: 28.3%; ≥41: 71.7% Controls: <41: 86.7%; ≥41: 13.3% mean ± SD total sleep time (hours) Cases: 8.58 ± 0.96 Controls: 9.38 ± 0.88 (sleep time sig. different between Cases and Controls) In DEF participants Total score Cases: 46.43 ± 8.04; sig Controls: 37.56 ± 2.80; sig Bedtime resistance Cases: 10.17 ± 2.66; sig Controls: 7.21 ± 0.75; NS Sleep-onset delay Cases: 1.82 ± 0.80; sig Controls: 1.08 ± 0.36; sig Sleep duration Cases: 4.70 ± 0.95; sig Controls: 4.18 ± 0.90; sig Sleep anxiety Cases: 6.78 ± 2.35; sig Controls: 4.05 ± 0.4; sig Night wakings Cases: 4.24 ± 1.59; sig Controls: 3.32 ± 0.62; sig Parasomnias Cases: 8.75 ± 1.84; sig Controls: 7.16 ± 0.44; sig Sleep-disordered breathing Cases: 3.41 ± 059; sig Controls: 3.40 ± 0.76; sig Daytime sleepiness Cases: 10.17 ± 1.93; NS Controls: 9.21 ± 1.08; sig Total sleep time Cases: 8.63 ± 0.85; sig Controls: 9.29 ± 0.89; sig	VDS may be beneficial in ASD patients and healthy individuals with sleep disturbances
Arico [38], Italy	RLS severity: IRLS-RS	10.3	30.4 (no information on statistical significance)	19.8	8.6 (no information on statistical significance)	VDS has a therapeutic effect in decreasing RLS severity
*Pre–post study, analyzed retrospectively as a case series*						
Sleep quality						
Huang [42], USA	Sleep quality: PSQI Sleep latency: #2 “how long has it usually taken you to fall asleep each night” of the PSQI Sleep duration: #4 “how many hours of actual sleep did you get at night” of the PSQI Sleep efficiency: #4 + #1 “what time have you usually gone to bed at night” + #3 “what time have you usually gotten up in the morning” of the PSQI	Total: 18.57 ± 5.42 INS: 22.73 ± 1.83 DEF: 13.77 ± 3.94	Total: 26.00 ± 8.38 (sig. increase) INS: 29.60 ± 11.67 DEF: 24.00 ± 5.79	Global PSQI score Total: 13.46 ± 4.92 INS: 12.27 ± 5.55 DEF: 14.85 ± 3.83 Sleep latency (min) Total: 67.22 ± 56.13 INS: 41.61 ± 48.21 DEF: 94.81 ± 52.15 Sleep duration (h) Total: 4.59 ± 1.84 INS: 5.33 ± 1.88 DEF: 3.73 ± 1.41 Sleep efficiency (%) Total: 59.79 ± 25.31 INS: 66.97 ± 23.7 DEF: 52.61 ± 25.70	Global PSQI score Total: 12.22 ± 4.61 (sig. decrease) INS: 11.29 ± 4.66 DEF: 13.23 ± 4.51 (sig. decrease) Sleep latency (min) Total: 57.86 ± 44.03 (sig. decrease) INS: 39.83 ± 39.05 DEF: 78.65 ± 41.33 (sig. decrease) Sleep duration (h) Total: 5.30 ± 1.57 (sig. decrease) INS: 5.90 ± 1.55 (sig. decrease) DEF: 4.62 ± 1.33 (sig. decrease) Sleep efficiency (%) Total: 66.62 ± 18.61 (sig. increase) INS: 70.34 ± 17.76 DEF: 62.33 ± 19.34 (sig. increase)	Sig. improvement in overall sleep quality, sleep latency, sleep duration, and sleep efficiency in veterans with multiple areas of chronic pain with VDS (after controlling for potential confounders improvement in sleep efficiency became borderline significant) The magnitudes of sleep improvement in latency, duration, and efficiency were all larger in the DEF subgroup; however, the difference in improvements between the subgroups was NS except for sleep latency

I: intervention; C: control; PSQI: Pittsburgh Sleep Quality Index; NR: not reported; INS: insufficient; DEF: deficient; VDS: vitamin D supplementation; 25(OH)D: 25-hydroxyvitamin D; SD: Standard Deviation; NS: Not Significant; sig. Significant; IU: International Unit; ESS: Epworth Sleepiness Scale; NR: not reported; RLS: restless legs syndrome; IRLSSG: International Restless Legs Syndrome Study Group; CI: confidence interval; IRLS-RS: International Restless Legs Syndrome Rating Scale; ADHD: Attention Deficit Hyperactivity Disorder; IQR: Interquartile Range; ASD: Autism Spectrum Disorder; CSHQ: Children’s Sleep Habits Questionnaire IRLS-RS: International Restless Legs Syndrome Rating Scale; IRLSSG: International Restless Legs Syndrome Study Group; CSHQ: Children’s Sleep Habits Questionnaire; PHQ-8 Item: The 8 item Patient Health Questionnaire depression scale; NA: Not Applicable; SE: Standard Error.

## Supplementary Materials

The following supporting information can be downloaded at: https://www.mdpi.com/article/10.3390/nu14051076/s1, S1,S2: Search strategy.
