# Supplementary material for: Vitamin D Supplementation and Sleep: A Systematic Review and Meta-Analysis of Intervention Studies
Source: Nutrients. 2022 Mar 3;14(5):1076. doi: 10.3390/nu14051076 (PMC8912284; doi:10.3390/nu14051076)
Supplement: Supplementary file 1 [file nutrients-14-01076-s001.zip › nutrients-1563453-supplementary.pdf]

Supplement 1: Search strategy

Medline

Database: Ovid MEDLINE(R) and Epub Ahead of Print, In-Process, In-Data-Review & Other Non-Indexed Citations and Daily <1946 to April 30, 2021>

Search Strategy:

----

1 exp Vitamin D/ or Vitamin D Deficiency/ (64926)

2 ("1406-16-2" or "vitamin d" or vitamind or vitamin-d).mp. (76478)

3 ("21343-40-8" or "25 hydroxycalciferol" or "25 hydroxyergocalciferol" or "25 hydroxyvitamin d 2" or "25 hydroxyvitamin d2" or "25-hydroxycalciferol" or "25-hydroxyergocalciferol" or "25-hydroxyvitamin d 2" or "25-hydroxyvitamin d2" or "9,10-secoergosta-5,7,10(19),22-tetraene-3 beta,25-diol" or ercalcidiol?).mp. (1072)

4 (Dihydrotachysterol or "at 10" or at-10 or at10 or calcamine or dihydrotachysterin or r5lm3h112r or tachystin or "67-96-9 (dihydrotachysterol)").mp. (93488)

5 ("50-14-6 (vitamin d 2)" or calciferols or ergocalciferol? or vs041h42xc or (vitamin adj1 ("d2" or "d 2"))).mp. (4156)

6 ("(24r)-24,25-dihydroxyvitamin d3" or "24,25 dihydroxycholecalciferol" or "24,25 dihydroxyvitamin d 3" or "24,25 dihydroxyvitamin d3" or "24,25-dihydroxycholecalciferol" or "24,25-dihydroxyvitamin d 3" or "24,25-dihydroxyvitamin d 3, (3beta,5z,7e,24r)-isomer" or "24,25-dihydroxyvitamin d3" or "24r,25 dihydroxycholecalciferol" or "24r,25-dihydroxycholecalciferol" or "40013-87-4" or "55721-11-4 ((3beta,5z,7e,24r)-isomer)" or "dihydroxyvitamin d3, 24,25").mp. (1125)

7 (Tirocal or soltriol or sitriol or silkis or rocaltrol or renatriol or osteotriol or "mc1288" or mc-1288 or "mc 1288" or calcitriol or fxc9231jvh or decostriol or "d3, 1,25-dihydroxy-20-epi-vitamin" or "d3, 1,25-dihydroxyvitamin" or "d3, 1 alpha,25-dihydroxyvitamin" or calcijex or bocatriol or "32222-06-3 (calcitriol)" or "20-epi-1alpha,25-dihydroxycholecaliferol" or "1,25-dihydroxy-20-epi-vitamin d3" or "1,25-dihydroxyvitamin d3" or "1,25-dihydroxycholecalciferol" or "1,25(oh)2-20epi-d3" or "1,25 dihydroxy 20 epi vitamin d3" or "1,25 dihydroxyvitamin d3" or "1,25 dihydroxycholecalciferol" or "1 alpha,25-dihydroxyvitamin d3" or "1 alpha,25-dihydroxycholecalciferol" or "1 alpha,25 dihydroxyvitamin d3" or "1 alpha, 25-dihydroxy-20-epi-vitamin d3").mp. (24070)

8 (Dihydroxycholecalciferols or "dihydroxyvitamins d").mp. (2110)

9 (t0wxw8f54e or p6yz13c99q or hidroferol or dedrogyl or calderol or calcifediol or calcidiol or "73809-05-9 ((3 alpha,5z,7e)-isomer)" or "36149-00-5 ((3 beta,5e,7e)-isomer)" or "25-hydroxyvitamin d3" or "25-hydroxyvitamin d 3" or "25-hydroxycholecalciferol" or "25 hydroxycholecalciferol" or "25 hydroxyvitamin d 3" or "25 hydroxyvitamin d3" or "73809-05-9 ((3 alpha,5z,7e)-isomer)" or "36149-00-5 ((3 beta,5e,7e)-isomer)" or "25-hydroxyvitamin d3" or "25-hydroxyvitamin d 3").mp. (7477)

10 (Hydroxycholecalciferol? or "hydroxyvitamins d").mp. (4674)

11 ("vitamin d3" or "vitamin d 3" or cholecalciferol? or calciol or "67-97-0 (cholecalciferol)" or "1c6v77qf41" or "(3 beta,5z,7e)-9,10-secocholesta-5,7,10(19)-trien-3-ol").mp. (17609)

12 1 or 2 or 3 or 4 or 5 or 6 or 7 or 8 or 9 or 10 or 11 (186780)

13 (Rickets or rachitis or rachitid?s or Osteomalacia? or ckd-mbd or "chronic kidney disease mineral and bone disorder" or "chronic kidney disease-mineral and bone disorder" or (osteodystroph* adj1 renal) or "hypophosphatemia, x linked" or "hypophosphatemia, x-linked").mp. (19693)

14 exp Vitamin D/ or exp Vitamin D Deficiency/ (73434)

15 12 or 13 or 14 (197714)

16 exp Sleep/ (83092)

17 (Dream* or nightmare? or night-mare? or (night adj1 terror*) or "pavor nocturnus").mp. (17137)

18 (sleep* or asleep or a-sleep or "n1-sleep" or "n2-sleep" or "nrem stage 1" or "nrem stage 2" or "nrem stage 3").mp. (221749)

19 ("rapid eye" adj1 movement*).mp. (10869)

20 exp Sleep Wake Disorders/ or APNEA/ (100948)

21 ((subwakefullness adj1 syndrome?) or Dyssomnia? or (("eating drinking" or eating-drinking) adj1 syndrome?) or (disturbed adj2 rhythm*) or (jet adj lag*) or "time zone change syndrome" or "time zone syndrome" or hypersomnia? or hypersomnolence or doess or ((daytime or excessive) adj1 somnolence) or kleine-levin or "kleine levin" or narcolep* or (gelineaus* adj syndrome) or CATALEP* or cataplex* or ((henneberg or tonelessness) adj1 syndrome?) or (nocturna* adj3 (bruxism or wandering or syndrome? or disorder? or cramp? or jactatio or banging)) or "periodic limb movement disorder" or (restless adj leg*) or ((willis or wittmaack) adj1 ekbom) or Apnea or osahs or insomnia? or (early adj1 awakening) or Parasomnia? or dystonia? or (rem adj2 disorder?) or (confusional adj1 arousal) or somnambulism or somnolescent or sopor).mp. (119670)

22 16 or 17 or 18 or 19 or 20 or 21 (281895)

23 12 and 22 (1235)

24 15 and 22 (1255)

****

Embase

No.	Query	Results
#37	#14 AND #36	4215
#36	#15 OR #16 OR #17 OR #18 OR #19 OR #20 OR #21 OR #22 OR #23 OR #24 OR #25 OR #26 OR #27 OR #28 OR #29 OR #30 OR #31 OR #32 OR #33 OR #34 OR #35	520158
#35	(confusional NEAR/1 arousal):ti,ab,kw	67
#34	(rem NEAR/2 disorder$):ti,ab,kw	765
#33	(early NEAR/1 awakening):ti,ab,kw	241
#32	((willis OR wittmaack) NEAR/1 ekbom):ti,ab,kw	329
#31	(nocturna* NEAR/3 (bruxism OR wandering OR syndrome$ OR disorder$ OR cramp$ OR jactatio OR banging)):ti,ab,kw	1562
#30	((henneberg OR tonelessness) NEAR/1 syndrome$):ti,ab,kw	0
#29	((daytime OR excessive) NEAR/1 somnolence):ti,ab,kw	1643
#28	(disturbed NEAR/2 rhythm*):ti,ab,kw	451
#27	(subwakefullness NEAR/1 syndrome$):ti,ab,kw	0
#26	(gelineaus* NEXT/1 syndrome):ti,ab,kw	0
#25	(jet NEXT/1 lag*):ti,ab,kw	1362
#24	('eating drinking' NEAR/1 syndrome$):ti,ab,kw	6
#23	(restless NEXT/0 leg*):ti,ab,kw	0
#22	dyssomnia$:ti,ab,kw OR 'time zone change syndrome':ti,ab,kw OR 'time zone syndrome':ti,ab,kw OR hypersomnia$:ti,ab,kw OR hypersomnolence:ti,ab,kw OR doess:ti,ab,kw OR 'kleine levin':ti,ab,kw OR narcolep*:ti,ab,kw OR catalep*:ti,ab,kw OR cataplex*:ti,ab,kw OR 'periodic limb movement disorder':ti,ab,kw OR apnea:ti,ab,kw OR osahs:ti,ab,kw OR insomnia$:ti,ab,kw OR parasomnia$:ti,ab,kw OR dystonia$:ti,ab,kw OR somnambulism:ti,ab,kw OR somnolescent:ti,ab,kw	149962
#21	'apnea'/exp	28857
#20	'sleep disorder'/exp OR 'apnea'/exp	299178
#19	('rapid eye' NEAR/1 movement*):ti,ab,kw	13876
#18	sleep*:ti,ab,kw OR asleep:ti,ab,kw OR 'a sleep':ti,ab,kw OR 'n1-sleep':ti,ab,kw OR 'n2-sleep':ti,ab,kw OR 'nrem stage 1':ti,ab,kw OR 'nrem stage 2':ti,ab,kw OR 'nrem stage 3':ti,ab,kw	302314
#17	'night-mare$':ti,ab,kw OR ((night NEAR/1 terror*):ti,ab,kw)	427
#16	dream*:ti,ab,kw OR nightmare$:ti,ab,kw OR 'pavor nocturnus':ti,ab,kw	20846
#15	'sleep'/exp	256353
#14	#12 OR #13	295471
#13	rickets:ti,ab,kw OR rachitis:ti,ab,kw OR rachitid?s:ti,ab,kw OR osteomalacia?:ti,ab,kw OR 'ckd mbd':ti,ab,kw OR 'chronic kidney disease mineral and bone disorder':ti,ab,kw OR 'chronic kidney disease-mineral and bone disorder':ti,ab,kw OR ((osteodystroph* NEAR/1 renal):ti,ab,kw) OR 'hypophosphatemia, x linked':ti,ab,kw OR 'hypophosphatemia, x-linked':ti,ab,kw	14223
#12	#1 OR #2 OR #3 OR #4 OR #5 OR #6 OR #7 OR #8 OR #9 OR #10 OR #11	289306

#11	'vitamin d3':ti,ab,kw OR 'vitamin d 3':ti,ab,kw OR cholecalciferol$:ti,ab,kw OR calciol:ti,ab,kw OR '67-97-0 (cholecalciferol)':ti,ab,kw OR '1c6v77qf41':ti,ab,kw OR '(3 beta,5z,7e)-9,10-secocholesta-5,7,10(19)-trien-3-ol':ti,ab,kw	14129
#10	hydroxycholecalciferol$:ti,ab,kw OR 'hydroxyvitamins d':ti,ab,kw	1779
#9	t0wxw8f54e:ti,ab,kw OR p6yz13c99q:ti,ab,kw OR hidroferol:ti,ab,kw OR dedrogyl:ti,ab,kw OR calderol:ti,ab,kw OR calcifediol:ti,ab,kw OR calcidiol:ti,ab,kw OR '25-hydroxycholecalciferol':ti,ab,kw OR '25 hydroxycholecalciferol':ti,ab,kw OR '25 hydroxyvitamin d 3':ti,ab,kw OR '25 hydroxyvitamin d3':ti,ab,kw OR '73809-05-9 ((3 alpha,5z,7e)-isomer)':ti,ab,kw OR '36149-00-5 ((3 beta,5e,7e)-isomer)':ti,ab,kw OR '25-hydroxyvitamin d3':ti,ab,kw OR '25-hydroxyvitamin d 3':ti,ab,kw	3891
#8	dihydroxycholecalciferols:ti,ab,kw OR 'dihydroxyvitamins d':ti,ab,kw	14
#7	tirocal:ti,ab,kw OR soltriol:ti,ab,kw OR sitriol:ti,ab,kw OR silkis:ti,ab,kw OR rocaltrol:ti,ab,kw OR renatriol:ti,ab,kw OR osteotriol:ti,ab,kw OR 'mc1288':ti,ab,kw OR 'mc 1288':ti,ab,kw OR calcitriol:ti,ab,kw OR fxc9231jvh:ti,ab,kw OR decostriol:ti,ab,kw OR 'd3, 1,25-dihydroxy-20-epi-vitamin':ti,ab,kw OR 'd3, 1,25-dihydroxyvitamin':ti,ab,kw OR 'd3, 1 alpha,25-dihydroxyvitamin':ti,ab,kw OR calcijex:ti,ab,kw OR bocatriol:ti,ab,kw OR '32222-06-3 (calcitriol)':ti,ab,kw OR '20-epi-1alpha,25-dihydroxycholecaliferol':ti,ab,kw OR '1,25-dihydroxy-20-epi-vitamin d3':ti,ab,kw OR '1,25-dihydroxyvitamin d3':ti,ab,kw OR '1,25-dihydroxycholecalciferol':ti,ab,kw OR '1,25(oh)2-20epi-d3':ti,ab,kw OR '1,25 dihydroxy 20 epi vitamin d3':ti,ab,kw OR '1,25 dihydroxyvitamin d3':ti,ab,kw OR '1,25 dihydroxycholecalciferol':ti,ab,kw OR '1 alpha,25-dihydroxyvitamin d3':ti,ab,kw OR '1 alpha,25-dihydroxycholecalciferol':ti,ab,kw OR '1 alpha,25 dihydroxyvitamin d3':ti,ab,kw OR '1 alpha, 25-dihydroxy-20-epi-vitamin d3':ti,ab,kw	11030
#6	'(24r)-24,25-dihydroxyvitamin d3':ti,ab,kw OR '24,25 dihydroxycholecalciferol':ti,ab,kw OR '24,25 dihydroxyvitamin d 3':ti,ab,kw OR '24,25 dihydroxyvitamin d3':ti,ab,kw OR '24,25-dihydroxycholecalciferol':ti,ab,kw OR '24,25-dihydroxyvitamin d 3':ti,ab,kw OR '24,25-dihydroxyvitamin d 3, (3beta,5z,7e,24r)-isomer':ti,ab,kw OR '24,25-dihydroxyvitamin d3':ti,ab,kw OR '24r,25 dihydroxycholecalciferol':ti,ab,kw OR '24r,25-dihydroxycholecalciferol':ti,ab,kw OR '40013-87-4':ti,ab,kw OR '55721-11-4 ((3beta,5z,7e,24r)-isomer)':ti,ab,kw OR 'dihydroxyvitamin d3, 24,25':ti,ab,kw	307
#5	'50-14-6 (vitamin d 2)':ti,ab,kw OR calciferols:ti,ab,kw OR ergocalciferol$:ti,ab,kw OR vs041h42xc:ti,ab,kw OR ((vitamin NEAR/1 ('d2' OR 'd 2')):ti,ab,kw)	3438
#4	dihydrotachysterol:ti,ab,kw OR 'at 10':ti,ab,kw OR at10:ti,ab,kw OR calcamine:ti,ab,kw OR dihydrotachysterin:ti,ab,kw OR r5lm3h112r:ti,ab,kw OR tachystin:ti,ab,kw OR '67-96-9 (dihydrotachysterol)':ti,ab,kw	114742
#3	'21343-40-8':ti,ab,kw OR '25 hydroxycalciferol':ti,ab,kw OR '25 hydroxyergocalciferol':ti,ab,kw OR '25 hydroxyvitamin d 2':ti,ab,kw OR '25 hydroxyvitamin d2':ti,ab,kw OR '25-hydroxycalciferol':ti,ab,kw OR '25-hydroxyergocalciferol':ti,ab,kw OR '25-hydroxyvitamin d 2':ti,ab,kw OR '25-hydroxyvitamin d2':ti,ab,kw OR '9,10-secoergosta-5,7,10(19),22-tetraene-3 beta,25-diol':ti,ab,kw OR ercalcidiol$:ti,ab,kw	304
#2	'1406-16-2':ti,ab,kw OR vitamind:ti,ab,kw OR 'vitamin d':ti,ab,kw	105872
#1	'vitamin d'/exp OR 'vitamin d deficiency'/de	160393

****

The Cochrane Library

ID Search Hits
#1 MeSH descriptor: [Vitamin D] explode all trees 5567
#2 MeSH descriptor: [Vitamin D Deficiency] explode all trees 1497
#3 ((vitamin-d OR "vitamin d" OR ercalcidiol? OR vitamind)):ti,ab,kw 12467
#4 ((Dihydrotachysterol OR calcamine OR dihydrotachysterin OR r5lm3h112r OR tachystin)):ti,ab,kw 37
#5 (calciferols OR ergocalciferol? OR vs041h42xc):ti,ab,kw 1713
#6 (Tirocal OR soltriol OR sitriol OR silkis OR rocaltrol OR renatriol OR osteotriol OR “mc1288” OR mc-1288 OR “mc 1288” OR calcitriol OR fxc9231jvh OR decostriol OR calcijex OR bocatriol):ti,ab,kw 1869
#7 (Dihydroxycholecalciferols OR “dihydroxyvitamins d”):ti,ab,kw 70
#8 (t0wxw8f54e OR p6yz13c99q OR hidroferol OR dedrogyl OR calderol OR calcifediol OR calcidiol OR Hydroxycholecalciferol? OR “hydroxyvitamins d” OR “vitamin d3” OR “vitamin d 3” OR cholecalciferol? OR calciol OR 1c6v77qf41):ti,ab,kw 5785
#9 ((osteodystroph* NEAR/0 renal)):ti,ab,kw 0
#10 (Rickets OR rachitis OR rachitid?s OR Osteomalacia? OR ckd-mbd OR “chronic kidney disease mineral and bone disorder” OR "hypophosphatemia, x linked"):ti,ab,kw 691
#11 #1 OR #2 OR #3 OR #4 OR #5 OR #6 OR #7 OR #8 OR #9 OR #10 14761
#12 MeSH descriptor: [Sleep] explode all trees 5805
#13 MeSH descriptor: [Sleep Wake Disorders] explode all trees 8398
#14 MeSH descriptor: [Apnea] this term only 989
#15 ((Dream* OR nightmare? OR night-mare? OR (night NEAR/1 terror*) OR “pavOR nocturnus”)):ti,ab,kw 1818
#16 ((sleep* OR asleep OR "n1-sleep" OR "n2-sleep" OR "nrem stage 1" OR "nrem stage 2" OR "nrem stage 3")):ti,ab,kw 41600
#17 ((“rapid eye” NEAR/1 movement*)):ti,ab,kw 916
#18 (((subwakefullness NEAR/1 syndrome?) OR Dyssomnia? OR ((eating NEXT/1 drinking) NEAR/1 syndrome?) OR (disturbed NEAR/2 rhythm*) OR (jet NEXT/1 lag*) OR “time zone change syndrome” OR “time zone syndrome” OR hypersomnia? OR hypersomnolence OR doess OR ((daytime OR excessive) NEAR/1 somnolence) OR kleine-levin OR “kleine levin” OR narcolep* OR (gelineaus* NEXT/1 syndrome) OR CATALEP* OR cataplex* OR ((henneberg OR tonelessness) NEAR/1 syndrome?) OR (nocturna* NEAR/3 (bruxism OR wandering OR syndrome? OR disorder? OR cramp? OR jactatio OR banging)) OR “periodic limb movement disorder” OR (restless NEXT/1 leg*) OR ((willis OR wittmaack) NEAR/1 ekbom) OR Apnea OR osahs OR insomnia? OR (early NEAR/1 awakening) OR Parasomnia? OR dystonia? OR (rem NEAR/2 disorder?) OR (confusional NEAR/1 arousal) OR somnambulism OR somnolescent)):ti,ab,kw 23816
#19 {OR #12-#18} 52077
#20 #11 AND #19 161
